# Evaluating Bias in Social Media Research Using #Sunscreen Content on Instagram Reels

**DOI:** 10.2196/76829

**Published:** 2025-11-05

**Authors:** Silvija Milanovic, Chelsea Rosen, Taylor Gray

**Affiliations:** 1 College of Medicine, University of Florida, 1104 Newell Dr, Gainesville, FL, 32601, United States, 1 352-273-7986; 2 Department of Dermatology - Jacksonville, University of Florida, Jacksonville, FL, United States

**Keywords:** social media, dermatology, health education, algorithms, information dissemination

## Abstract

This cross-sectional content analysis found that Instagram’s hashtag-based reels display consistent dermatologic content regardless of user engagement history, supporting the use of hashtags as an objective and reproducible tool for social media research in dermatology.

## Introduction

Social media has become a source of medical information for the general public, with Instagram and TikTok being among the most influential apps [1-4]. In response to the increasing presence of dermatology-related content, dermatologists have been encouraged to engage in social media to decrease misinformation [1-4]. As dermatology-related content grows on social media, more research is being done to assess its quality [1]. Concerns have been raised about the reliability and reproducibility of social media research, particularly due to the personalized nature of platform algorithms [1]. A 2024 study by Druskovich and Landriscina [1] highlighted how TikTok’s content curation can introduce bias and inconsistency into dermatology research.

This study aimed to assess whether account bias affects the type of dermatologic information presented in social media videos.

## Methods

### Overview

We used Instagram reels rather than TikTok amid increasing scrutiny of TikTok’s data privacy practices and the potential for a national ban in the United States. We compared Instagram reels videos between two user accounts: one with a biased profile based on prior dermatology engagement, and a new account with no activity or history. The biased profile was an existing Instagram account that had engaged extensively with dermatology-related content. This account followed multiple dermatologists and dermatology organizations and had previously liked, commented on, and shared dermatology posts, thereby establishing a history of dermatology-related activity.

For each account, the first 100 reels displayed under the hashtag #sunscreen were saved to prevent future content influence, resulting in a total of 200 videos. Each video was evaluated and categorized by creator type (dermatologist, non-dermatologist physician, esthetician, beauty blogger, etc) and content type (educational, advertisement, entertainment, etc). Creators were classified as dermatologists if they were verified to be board-certified through online professional profiles, institutional websites, or board certification databases. Engagement metrics, including views, likes, and comments, were recorded. By analyzing both content and engagement metrics, we sought to determine whether Instagram’s hashtag-based video results are influenced by user history or whether they offer an objective method for dermatology social media research. Data analysis was performed using R (version 4.3.1; R Foundation for Statistical Computing) with a significance value set to *P*<.05.

### Ethical Considerations

This study was deemed exempt from ethics approval as it did not involve human participants or identifiable private information. The research was conducted in accordance with the ethical standards outlined in the U.S. Department of Health and Human Services, Code of Federal Regulations, Title 45, Part 46 [5]. All data analyzed were publicly available and fully deidentified prior to analysis, ensuring no individual could be identified directly or indirectly.

## Results

Our study found there were no statistically significant differences in the video results between the two accounts. A χ^2^ test assessing differences in content categories and creator types showed no significant variation between accounts (*χ²*_₆_ = 7.6, *P* = 0.3; Table 1). A Monte-Carlo chi-squared test evaluating video types similarly found no significant differences (*χ*²_₆_=19.9, *P*=0.8; Table 2). Engagement metrics for views, likes, and comments across all creator types were compared using two-sample *t* tests, and all *P* values exceeded 0.05. A paired *t* test comparing video engagement, calculated by adding the likes and comments and dividing by the view number, showed no difference between accounts (*P*=0.42; Table 1). These findings suggest that the Instagram reels hashtag system displays dermatologic content consistently and reproducibly, regardless of account history or engagement.

**Table 1.** Video engagement by creator type for reels found on a biased profile based on prior dermatology engagement and a new account with no activity or history.

	Number of videos		Average views		Average likes		Average comments		Average engagement rate	
Video Creator	Biased	Unbiased	Biased	Unbiased	Biased	Unbiased	Biased	Unbiased	Biased	Unbiased
Dermatologist	17	14	1,950,376	3,814,600	25,112	46,071	578	2899	1.52%	1.58%
Non-dermatologist physician	4	4	1,233,550	975,325	14,086	12,554	482	562	1.47%	1.26%
Nurse practitioner	1	1	9,200,000	10,400,000	428,000	480,000	1008	1087	4.66%	4.63%
Esthetician	1	8	67,100	13,938,500	334	38,620	63	361	0.59%	1.25%
Beauty blogger	59	58	3,047,975	2,166,548	47,610	51,883	664	2169	2.89%	3.11%
Skincare company	10	5	3,734,560	5,362,400	60,578	96,881	396	386	1.94%	2.53%
Other	9	11	2,769,867	3,137,000	75,329	76,686	811	859	13.08%	1.99%

**Table 2.** Video type by creator type for number of reels found on a biased profile based on prior dermatology engagement and a new account with no activity or history.

	Educational		Personal experience		Business/Advertisement		Entertainment/Humor		Clinical demonstration	
Video creator	Biased	Unbiased	Biased	Unbiased	Biased	Unbiased	Biased	Unbiased	Biased	Unbiased
Dermatologist	10	6	1	2	4	4	2	2	0	0
Non-dermatologist physician	3	3	1	1	0	0	0	0	0	0
Nurse practitioner	0	0	0	0	0	0	1	1	0	0
Esthetician	0	2	0	3	0	2	1	0	0	1
Beauty blogger	9	9	22	21	23	20	5	6	0	2
Skincare company	0	0	0	1	6	2	3	2	1	0
Other	2	4	0	1	1	0	3	2	3	4

## Discussion

These results challenge assumptions that social media research is inherently flawed due to account engagement history [1]. While Instagram curates personalized content based on user interaction, the hashtag system appears to present the same type of information regardless of account history. This has meaningful implications for research methodology, where Instagram hashtags may provide an objective tool for studying dermatologic content online, supporting more reproducible and rigorous investigations.

Our study is limited as it reflects a single time point and one hashtag on one platform. Future work should evaluate the longitudinal stability of Instagram’s algorithm, explore other hashtags, and compare results across broader time frames. Additionally, while the hashtag system offers a consistent sampling method for research, it may not fully reflect the way users typically consume content. Many users do not actively search via hashtags, and some creators, especially those with large followings, may not rely on hashtags for engagement, meaning their content may be underrepresented in hashtag-based analyses. Despite this, the hashtag system provides a practical and reproducible approach for evaluating dermatologic content for research.

As patients continue to turn to social media for skin health information, understanding how content is delivered empowers dermatologists to better counsel patients on how to navigate these platforms critically. These findings may reassure clinicians that accurate, evidence-based content has the potential to compete with less credible sources, especially when paired with effective use of tools like hashtags. Our data show that dermatologists did not receive the highest engagement rate (Table 1), highlighting the need for more dermatologists to create engaging social media content. Rather than dismissing social media as a space dominated by misinformation, dermatologists can leverage these insights to engage with patients and encourage more informed digital consumption.
